# "It's in my life": the significance and consumption of lifestyle sports participation among Chinese youth

**DOI:** 10.3389/fspor.2025.1611549

**Published:** 2025-09-04

**Authors:** Longyin Chen, Jiru Guo, Juan Song

**Affiliations:** ^1^ School of Arts, Xi'an Physical Education University, Xi'an, China; ^2^ School of Economics and Management, Shanghai University of Sport, Shanghai, China; ^3^ School of Media and Art, Tianjin University of Sport, Tianjin, China

**Keywords:** lifestyle sports, young people, consumption practices, ultimate frisbee, China

## Abstract

**Introduction:**

Sports have become an indispensable part of daily life, and an increasing range of lifestyle sports, such as frisbee, flag football, and surfskate, are gradually becoming familiar to and popular among the Chinese youth. This study examines the consumption practices of Chinese youth in lifestyle sports through the analytical lens of Social Practice Theory, highlighting their distinctive cultural significance for this age group.

**Methods:**

This study utilized semi-structured interviews and participant observation to conduct research in Xi'an City, Shaanxi Province, China.

**Results:**

The findings revealed that participation in these sports serves not only as a form of consumption but also as a way for youth to break the discipline of life, shaping distinctions in taste while building tribalism and social interaction.

**Discussion:**

The study further explores the distinctive consumption traits of contemporary Chinese youth and underscore the social value of lifestyle sports. By situating these practices within the framework of Social Practice Theory, the study broadens the scope of sports consumption research and offers nuanced insight into youth behavior and consumption patterns amid China's ongoing social transformation.

## 1 Introduction

Since the 2022 Beijing Winter Olympics, China has experienced an unprecedented surge in what is commonly referred to as “lifestyle sports.” Unlike traditional competitive sports or state-led mass fitness initiatives, lifestyle sports emphasize personalization, aesthetic expression, and a departure from institutionalized structures. Typical examples include surfing, skateboarding, BMX riding, and mountain biking. As Wheaton (1) notes, lifestyle sports are not merely displays of physical prowess—they are deeply embedded in social and cultural meanings, encompassing identity, taste, style, and a sense of belonging. This broader conceptual framework enables trends in lifestyle sports in China, such as ultimate frisbee, flag football, and surfskate, to be categorized as part of the lifestyle sports spectrum. In the Chinese context, the grassroots origins, everyday relevance, and growing appeal of lifestyle sports have spurred a rapid expansion in self-media production, online discourse, and consumer demand for related products. Industry data show the frisbee industry core market reached 7.59 billion yuan in 2021 (2), while skateboard-related videos exceeded 18 billion views on TikTok in 2022 (3). Academic studies further highlight how lifestyle sports in China function as spaces for youth mental health, social interaction, and the negotiation of urban construction (4–6). These developments mirror, yet also diverge from, international trajectories of lifestyle sport participation.

International research on lifestyle sports has grown steadily in recent years, with a predominant focus on identity, social benefits, sport policy, and motivation. These works have occurred primarily in Western contexts (7–10). Lately, however, scholarly interest has expanded toward East Asia. Evers and Doering (11), or instance, examined how lifestyle sports in East Asian societies intersect with local social, political, cultural, and economic structures. In a similar vein, McDuie-Ra (61) investigated how skateboarding subcultures disrupt spatial norms and challenge modernization discourses by enabling youth to reimagine and repurpose urban environments. Further contributions have explored the inclusion of skateboarding in the Olympic Games as a lens through which to understand the tensions between grassroots youth culture and top-down forms of state governance (12, 13). Nevertheless, a significant gap persists: the everyday consumption practices of young people within lifestyle sports, along with their deeper cultural meanings, remain insufficiently examined, particularly amid the extensive social transformations taking place in contemporary China. In China, the world's largest developing nation, rapid economic, cultural, and social shifts have heralded a different cultural logic and practice in the field of sports consumption compared to those evident in the West. Influenced by globalization and economic progress, the consumption preferences of Chinese youths—symbolization, personalization, and socialization (14)—are mirrored in their engagement with lifestyle sports. This reflects not only personal insights but also broader societal changes. Therefore, this study primarily seeks to answer the following questions: 1. What are the unique psychological connotations driving the demand among the youth population for lifestyle sports consumption? and 2. what value might lifestyle sports bring to social development? Given this perspective, the conceptual framework of consumption sociology was deployed to illuminate the development and implications of daily youth participation in lifestyle sports. This research not only updates traditional perceptions of sports consumption and broadens the scope of sports participant analysis, but it also serves as a Chinese case study for the international promotion and dissemination of lifestyle sports.

## 2 Previous research

### 2.1 Existing research and expansion

Academic research on lifestyle sports has gradually emerged since the late 20th century, undergoing a theoretical evolution from “marginal subculture” to “everyday cultural practice”. Foundational scholars such as Iain Borden, Becky Beal, and Belinda Wheaton were pivotal in laying the groundwork for this field. One defining feature of lifestyle sports is their relative disregard for traditional social hierarchies. Factors such as family background, economic status, or the financial cost of participation are de-emphasized. Instead, subcultural capital is earned through skill, sustained commitment, and personal effort (1, 15). A second key feature is their emphasis on performative expression (16). These sports are deeply intertwined with their environments and diverge from the conventional logic of “achievement sport culture”. (16). For instance, drawing on Lefebvre's spatial theory, Borden (17) framed skateboarding as a form of critique against capitalist urban space. He argued that the resurgence of street skateboarding in the 1990s constituted a performative act of resistance, challenging the spatial, social, and temporal logics embedded in capitalist urban life. Third, lifestyle sports are participant-driven. They operate with minimal formal regulation, and institutional structures—if present—are often loose and informally defined (18). Participants frequently position themselves in contrast to conventional athletes (19), adopting a cultural stance that resists bureaucratic norms and competitive orthodoxies. These studies lay the groundwork for the basic theoretical paradigm of lifestyle sports: it is not merely physical activity, but a cultural practice that encompasses aesthetics, emotion, and spatial politics. It challenges the traditional research focus of sports sociology, which centers on institutions, rules, and competition, and instead focuses on physical practice, performativity, and resistance (1).

In recent years, lifestyle sports have undergone notable shifts, driven by the rise of social media, commodification of urban space, and growing public awareness of health. As Li (20) observes, while these sports retain ties to the sub- and counter-cultural movements of the 1960s, their rebelliousness has shifted. No longer rooted in broad political agendas, they now reflect the everyday demands for personal autonomy and lifestyle choice (21). Meanwhile, scholars are increasingly arguing that lifestyle sports deserve greater attention in education as they may foster sustained participation more effectively than many traditional sports (22–24). Their motivations have also evolved. What once reflected opposition or assimilation now often forms a complex, mutually dependent relationship with commercial systems. Participants actively use mass media for self-promotion, while some communities adopt commercial models from the outset to organize youth engagement (25). Beyond individual expression, lifestyle sports also serve broader social functions. For example, the Singaporean government has rebranded skateboarding as a constructive activity, channeling youth energy away from delinquency while aligning with state-endorsed ideals of masculinity and academic excellence (26).

While the existing literature provides valuable insights into youth engagement in lifestyle sports, there is noticeable potential for further expansion. First, many studies portray youth participation as having a universal motive (5, 27), overlooking the inherent diversity and heterogeneity of their participation. Second, the sociocultural transformation mediated by day-to-day activities demands more intense scrutiny. Current research displays a deficient understanding of the subjective perspectives of youths regarding their choices and emotions. Third, most studies present merely straightforward accounts of facts. Adequate explorations into the transformative social value and underlying concepts brought about by youth engagement in lifestyle sports remain elusive. Owing to these observed deficiencies, this paper sought to extend the horizons of lifestyle sports and subculture studies through the adoption of a consumption practices standpoint.

### 2.2 Theoretical framework

In this study, Social Practice Theory was adopted to examine the consumption behaviors of young people in lifestyle sports and their broader social significance. Originally introduced to bridge the divide between structure- and agency-centered approaches in sociology, the concept of “practice” gained academic traction through the work of scholars such as Bourdieu and Giddens. Social Practice Theory centers its analysis not on individuals, norms, or institutions but on everyday practices as the core unit of sociological inquiry and intervention. Reckwitz (28) defines practice as a constellation of bodily and mental activities, material artifacts and their use, and background knowledge, which would include experience, emotions, and motivation. Expanding on this, Shove et al. (29) identify three key elements of practice: materials, competence, and meaning. In the context of sport, materials include equipment and physical infrastructure; competence refers to embodied skills and rule-based knowledge; and meaning encompasses symbolic value, identity, and aspirations. Additionally, Lefebvre contributes a complementary perspective by emphasizing “the moment”—a brief, disruptive experience that transcends the routine logic of daily life. These emotionally charged instances offer self-realization and rupture the alienation of repetition (30). When applied in the current study, this suggests that lifestyle sports are not merely habitual activities but can also serve as critical moments of personal agency and meaning-making in evolving social contexts.

The focus on everyday life in Social Practice Theory has made it highly influential in consumer sociology. As Warde (31) aptly states, “Consumption occurs within practice and is also for the sake of practice.” He emphasizes the role of routine, ritualistic, and repetitive activities in shaping consumption; this contrasts sharply with the consumer culture paradigm, which prioritizes conscious, symbolic, and meaning-driven actions. In practice theory, consumption is often guided by practical consciousness rather than deliberate reflection. It is not merely a vehicle for expressing subjective meaning but also a bodily activity embedded in material contexts and supported by specific tools (32). For example, in their study of Nordic walking, Shove et al. (33) found that the rise in this activity was not simply a matter of personal preference. Instead, it resulted from a convergence of factors: discourses promoting health, sustainability, and nature; the evolving interaction between producers and consumers; and technological improvements in walking poles. Cahill et al. (34) examined key elements involved in sports betting consumption, including smartphone apps, online communities, sports knowledge, betting strategies, and emotional engagement. Their analysis highlights how these components interact to shape user behavior, revealing how platformization not only structures betting practices but also contributes to detrimental outcomes.

In the context of lifestyle sports consumption, Social Practice Theory provides an effective analytical framework. For many young people, engaging in activities such as frisbee, flag football, and skateboarding is not merely a matter of selecting a sport; it involves ongoing participation, embodied performance, and meaning-making that unfold within the rhythms of daily life. These practices emerge from the integration of multiple elements, including equipment, social norms, codes of field etiquette, bodily techniques, cultural values, and digital platforms. Furthermore, to further illuminate the emotional and communal dimensions of lifestyle sports, this study draws on Maffesoli's (35) concept of neo-tribes, which explains how affective ties, shared rituals, and informal gatherings deepen group cohesion and, in turn, shape patterns of sport-related consumption.

In summary, this study applies the perspective of Social Practice Theory to examine the generative mechanisms and social significance of adolescents' participation in lifestyle sports. This approach broadens the scope of sports consumption research and provides a nuanced lens through which to understand the everyday lives of contemporary urban Chinese youth.

## 3 Method

This study combines descriptive and qualitative research to examine lifestyle sports in China, the aim being to understand the consumption practices of today's youth in these sports programs. For several reasons, ultimate frisbee, flag football, and surfskate were chosen as entry points for examining lifestyle sports. First, by virtue of its “low threshold,” ultimate frisbee has recently developed a larger participant base than other lifestyle sports in China, while governmental and private sports organizations have gradually realized the process from scratch. Second, in 2023, the International Olympic Committee voted to add flag football to the 2028 Los Angeles Olympic Games program, so this new item was deemed appropriate to study. Third, surfskate is a one-person sport that requires less space and reaches a wider audience than other one-person activities.

The research team conducted fieldwork from October 2022 to May 2023 in and around Xi'an, Shaanxi Province, China, focusing on the categories of lifestyle sports, spatial layout, and participant behavior and consumption. The city of Xi'an was chosen as the study area for three reasons. First, the range of lifestyle sports and coverage of various lifestyle sports in the city are comprehensive. These activities include hiking and cross-country (outdoor sports); ultimate frisbee and flag football (urban recreation sports); and kayaking and paddle boarding (aquatic sports). This wide range of sports enhances the reliability of the study. Second, the development of these sports is complete. In the case of ultimate frisbee, for example, there are early active frisbee players, new frisbee enthusiasts, official teams in colleges and universities, community training teams, and after-school interest classes in primary and secondary schools. Furthermore, Xi'an hosts China's first ultimate frisbee league (36). Fourthly, this city is inland in western China, so choosing this type of location made the study more representative at the national intermediate level. Evidence was obtained through a strategy combining in-depth interviews and participant observation. The researcher joined various clubs, obtained membership of different organizations, and met several respondents through long-term covert participant observation. Preliminary observations were made of the behaviors and activity process of the sports participants in their respective settings. After five months, with the consent of each club manager, the researcher transitioned to overt participant observation and systematically documented the findings.

In participant observation, the researcher alternates between the roles of “full participant” and “observer.” Initially, the researcher joined a club as a member, engaging in exercises, conversations, and inquiries with respondents. This method fostered empathetic understanding, enabling the researcher to grasp unique perspectives and viewpoints more effectively. However, as the involvement intensified, the researcher's subjective emotions, perspectives, and thought processes increasingly influenced the observations. To ensure the objectivity and accuracy of the observations, the researcher engaged in discussions with another researcher when each session had ended. Throughout these discussions, concerted efforts were made to “step out of the role,” with perspectives on the observed scenes and characters being reassessed and adjusted to enhance the rigor and reliability of the study. This paper incorporates in-depth interviews to determine the consumption habits, related experiences, and perspectives of individual lifestyle sports participants. Additionally, the interview questions were adapted based on emerging themes and clues obtained during the sessions, the aim being to probe more deeply and explore complex issues more thoroughly.

According to the requirements of qualitative research, to ensure reliability and validity, the two principles of sample sufficiency and information saturation proposed by Seidman (37) were followed when selecting interview subjects. First, a purposive sampling scheme was adopted. According to the purpose of the study and relevant theories in the sociology and demography of sport, age was taken as the main sampling criterion [the World Health Organization defines youths as those aged from 14 to 44 years old (38)]. The differences in players' experience in their sport (based on the number of years), education levels, and occupations were also taken into consideration because these aspects could enable comprehensive coverage of the different views, experiences, or groups of people in relation to the research questions. Meanwhile, the researcher ensured that the recruited respondents were willing to share their sports experiences and sufficiently talkative in order to obtain sufficiently rich information. Second, based on Seidman's principles, the researcher also continually assessed whether the information collected was saturated. The sample was considered adequate when the participant interviews had covered the topics and information needed to address the research questions, with no new insights or information provided in the follow-up interviews. The representative interviewees are displayed in Table 1 below.

**Table 1:** Basic information about the interviewees.

Interviewee	Gender	Age	Experience in the sport	Current occupation	Chosen emerging sport	Club
LLY	Male	26	6 months	Self-employed	Frisbee	YUF (lead manager)
LSLH	Male	36	1 year	Director, painter	Frisbee	PANNER (master, member of the Association)
SX	Male	38	8 months	Film culture company planning	Surfskate	PANNER (trainer)
HZZ	Female	28	4 years	University teacher	Surfskate	WILD
LM	Male	46	6 years	University teacher	Frisbee	V7 (instructors)
YXY	Female	22	8 months	Elementary school teacher	Flag Football	YUF
LJY	Female	19	4 months	Student	Flag Football	GEMRUN (administrator)
ZL	Male	29	7 months	Architectural program designer	Surfskate	GOGOCIUB
LW	Male	32	6 months	Programmer	Frisbee	CHILI OIL SPLASH
LP	Male	34	4 months	Freelancer	Flag Football	NFL FLAG
YX	Female	27	2 years	Salesman	Frisbee	BJ ONE

Thematic analysis was employed to interpret the data, following the procedures outlined by Braun (39) and Clarke (40). This process involved: 1. generating initial codes from the interview transcripts; 2. repeatedly reviewing and refining these codes to identify patterns of meaning; and 3. developing interpretive themes by situating these patterns within the analytical framework of Social Practice Theory and the cultural context of Chinese youth. After systematic sorting, four central themes were finally refined: 1. Breaking the Discipline of Life, capturing how lifestyle sports disrupt regimented routines; 2. Shaping Distinctions in Taste, reflecting the role of media and aesthetics in identity construction; 3. Emotional connection and maintenance, describing the formation of affective communities; and 4. Real social, highlighting the pursuit of authentic social connections. These themes are illustrated in the results section through selected narratives and representative participant perspectives. Ethical approval for the project was obtained from the first author's university to cover the study duration. Advice was received from the University Ethics Committee, and the consent form specified that the data would be kept confidential; that the interviewees would remain as anonymous as possible; and that the consent forms signed by the interviewees specified that any identifying information would not be used intentionally.

## 4 Results

### 4.1 Breaking the discipline of life

In daily life, one's energy and mechanisms are subject to constant, subtle control, which shapes and disciplines the human body (41). The sense of alienation in industrialized steel cities, modern rigid work environments, the erosion of leisure time, and the public safety restrictions imposed during epidemics have left the urban youth physically and mentally drained and constrained. Although brief moments of pan-entertainment offer marginal relief from the restrictions on free time and space imposed by the social system, the pursuit of physical and mental freedom remains a major need when subject to the pressures of disciplinary mechanisms (6). Participants in the lifestyle sport of frisbee described their engagement with the activity as a means to break free from the societal constraints imposed by hierarchical structures and normative organizations.

You may have a nine-to-five job in the metropolis, you may not have moved in a long time, or perhaps you've been confined at home for an extended period due to the pandemic. But now, playing frisbee, you'll feel a transformation—you'll rediscover the sensation of controlling your body and the vitality of being healthy, in a space that belongs solely to you. (Frisbee, LSLH)

At this juncture, the significance of movement is a practice of individual control of freedom, and the return of body and mind in the movement is itself the significance. Participants in lifestyle sports shed the alienation experienced in traditional workspaces, seeking and crafting moments of authenticity and control that liberate them from the monotony of daily life. In proposing the “Moments Theory”, Lefebvre emphasized that moments are linked to a need to transform everyday life and to organize, plan, and arrange it (30). Through moments, individuals can experience a freedom and passion that transcends the ordinary, resisting the mechanization and monotony of life. This is how surfskate enthusiasts describe their sport, as exemplified by this participant:

You can empty your mind while skating, simply enjoying the sensation of wind and “waves” on land. Whether it's fun or not, it feels like flying without leaving the ground. (Surfskate, SX)

Lifestyle sports disrupt the conventional routines of daily life and temporarily suspend traditional cultural norms. Engaging in these sports offers young people a psychological restoration and a physical escape from urban confines. But how does the “moment” experienced in this lifestyle sport differ from the equivalent in traditional sports? Multiple research subjects provided similar responses in this regard.

Life already has plenty of conventions and restrictions, and at the gym, the instructor will tell me again to raise which muscle, how to do [something]—I don't want more control. But frisbee is different; there's no referee, we set our own rules, there are no strict pacing requirements, and it also minimizes physical contact. This is what I want. (Frisbee, LW)

It's different from all the hobbies I've been exposed to before—music, dance, traditional competitive sports, etc. It's incredibly free. No one dictates my actions or objectives, and the inherent freedom and diversity are what I find most appealing. (Surfskate, HZZ)

The simplified rules, unrestricted movement, and spirit of inclusiveness inherent in lifestyle sports were found to distinguish them clearly from traditional sports. For example, in ultimate frisbee, suspected fouls are negotiated between the two parties, and they become disputes in which no intervention is required from other players or even the referee. The stipulation that athletes cannot move their feet when they receive the disc but are allowed the buffer pace of a quick stop and to hold the disc for a few seconds is unlike the strict pace requirements, fixed procedures, and physical techniques of traditional sports. Implicit in this is the lack of acceptance among lifestyle sports enthusiasts of the confines of traditional athletics, which can apply to the rules of a game, the definition of a program, and the regulation of the physical movements of male and female athletes. As a transient, anti-normative experience, the “moment” offered by lifestyle sports can expose and challenge the constraints of a disciplined society. This approach often uses physical activity as a means to alleviate the material and mental burdens of reality, aiming to achieve unparalleled relaxation and mental freedom. In this context, pure participation becomes the objective, with “flow” representing the ultimate experiential goal of this consumer practice. In her study on skiing, Thorpe (42) argues that lifestyle sports create “liminal spaces” where young people evade normative bodily control, reclaim agency, and experience immersion both emotionally and physically. These ideas resonate strongly with the narratives of freedom, bodily control, and self-imposed rules described above by Chinese youths. Yet important distinctions emerge. On the one hand, cumulative structural pressures—ranging from pandemic-induced spatial confinement to the “996” work regime—have made bodily liberation an increasingly urgent demand among the Chinese youth. On the other hand, their motivation is cultivated through platform discourse, brand marketing, and policy advocacy rather than the “anti-commercial,” “anti-institutional,” and “anti-cultural” milieus that nurture similar practices in the West.

### 4.2 Shaping distinctions in taste in a mediated society

Young people who have grown up in an era of material abundance seek to display their unique consumer insights and tastes, thereby distinguishing themselves from the general public in terms of consumer realms, perceptions, and styles (43, 44). This drive for distinction is also evident in the consumption practices of lifestyle sports. Here, however, symbolic meaning shifts from the quantity of consumption and traditional markers like equipment and clothing to the attitude toward participation, the commitment level, and the spiritual connotations of the sport itself. Emphasis is placed on tangible proofs or social media dissemination, such as taking photographs, recording videos, and uploading clips of participation onto online platforms. Since a sports experience is subjective and intangible, being retained only in memory and not easily visualized as symbols, these material proofs presented on social media enable the realization of differentiation.

More specifically, this symbol-driven realization of consumption is manifested in two ways. Firstly, participants achieve self-presentation and external validation through lifestyle sports. On one hand, fashion aesthetics have defined the commercial attributes of the female body, with a youthful, slender, and attractive image being favored. Ultimate frisbee offers a new arena for young women to showcase their physicality. On the other hand, participants take pride in their involvement in lifestyle sports, viewing it as a form of social capital to showcase to the outside world. One respondent shared their view on this:

Although my schedule is tight, I make it a point to get out every weekend to play a couple of games of flag football. It rejuvenates my weakened body and helps me manage life better; I can't function properly without a regular workout routine. I can take control of my body, and I'm proud to show it off. And of course, I'm going to tweet about it—otherwise who [would] know? (Flag football, LJY)

In many contemporary youth lifestyles, structural adjustments in industry have made sedentarism a common workplace posture. Additionally, the increased specialization of job roles and intense competition are contributing to rising levels of physical and mental stress among young people, thereby elevating the value placed on sports for achieving a healthy body. In that context, youths link lifestyle campaigns to ethical evaluations of their own willpower and time management skills. Whether it concerns a healthy body or a female body, the practice of sharing photographs and sports narratives online enriches their symbolic significance. This not only showcases physical health but also reflects the individual's mental positivity, confidence, and cheerfulness, aligning with broadly recognized societal values.

The second way in which this realization of consumption is manifested is the distinctive taste preferences showcased by lifestyle sports, such as being cool and hip. Respondent YXY characterized their switch from frisbee to flag football as driven by a desire to “do things differently from other people”.

Everyone was flocking to frisbee, and I disliked how it dominated my circle of friends, which made me reluctant to join. But when I discovered the flag football club, I thought it was incredibly cool at first glance. I'm going to [try it]! Also I quite enjoy this different feeling. (Flag football, YXY)

In this type of consumption practice, young people prioritize the relationship between themselves and the external environment, viewing their peers and media-influenced acquaintances as benchmarks for their actions. They prefer to follow current fashions and trends over traditional norms. The rise of symbol-driven consumption highlights the tendency among young people to follow in sports participation and lead in fashion trends. This phenomenon stems from not only contemporary youth's consumer preferences but also the historical and cultural context of the sports themselves. Originating in the 1960s, sports like ultimate frisbee and flag football began as youth subcultures characterized by a rebellious nature. The term “cool” emerged as an abstract summary of the attitudes prevalent among hippies and yuppies of the 1980s. It refers to and encapsulates a reductionist worldview that is indifferent to traditional social values and seeks simplicity, individuality, and enjoyment (45).

Being called ultimate (frisbee) [means] that I am different from them; I am above the rest. For example, it's frisbee without referees, like our slogan: Make love, not war. (Frisbee, LM)

Furthermore, in lifestyle sports communication, perceptions and behaviors influenced by a mediated society facilitate the popularity of sports programs. This is because sharing lifestyle sports experiences and posting updates on social media have become common practices. While traditional sports rely on physical participation or spectating (including online viewing) for communication, in lifestyle sports, every participant acts as a communicator, rapidly amplifying their sport's visibility and encouraging more people to join. This participation is then displayed and shared on various platforms, creating a cycle of visibility and engagement.

Frisbee caught my eye with its appeal; I saw it on Xiaohongshu,^1^ found it intriguing, and decided to try it out. It felt like something straight out of a movie, and there were many moments worth sharing. (Frisbee, YX)

### 4.3 Neo-tribalism in an individualized society

While the consumption practices of youth lifestyle sports are embodied in symbolic meaning, the “collective” aspect appears to predominantly retain the sense of community and emotional connection fostered by the lifestyle sport itself. As one interviewee remarked,

I came here [to the frisbee club] with no particular reason, just to play frisbee. I always feel that the popular sports are not quite the same nowadays; [there is] a feeling of unreality which will make you feel comfortable, and you will plan to come back again. (Frisbee, LLY)

In using the term “unreality” in relation to lifestyle sports, respondents referred to what they perceived as a purely emotional exchange. This stems from the fact that, for youths, relationships are often rooted in practical interests and goal-oriented interactions. More specifically, in contemporary China, political and economic reforms have introduced significant uncertainty, while rapid social mobility affects nearly every dimension of young people's lives (46). As a result, today's youth often experience growing feelings of loneliness and helplessness, akin to the “atom of isolation” (47). Consequently, social connections have shifted from traditional emotional and interpersonal bonds to more utilitarian, goal-driven relationships (48). In a sense, however, lifestyle sports have changed this by creating a specific atmosphere, feeling, and emotion in which individuals can gain a brief but real sense of belonging, freedom, and emotional response. This experience exemplifies what Maffesoli (35) describes as a key dimension of “neo-tribalism.” In highly individualized and institutionalized societies dominated by functional rationality, traditional groups—based on blood, class, and occupation—have gradually disintegrated. Yet the emotional need for community persists and is now reconstituted through new lifestyles and cultural consumption. Lifestyle sports thus serve as platforms for emotional sharing, offering participants a fleeting but profound sense of “unreality.”.

Immediately upon joining one activity, the researcher discerned among the members a psychological contract rooted in trust and inclusion, It was also characterized by an implicit agreement to respect personal privacy—including job, position, and salary—and maintain confidentiality. Everyone cooperated to maintain this tacit understanding. As one interviewee said after a session:

It's a much more pleasant atmosphere; they [other players] don't care about your identity [or] your family background. Everyone should have their own space, [and] sport should just be sport. (Flag football, YXY)

Moreover, sports equipment and skills were transformed into gifts. This occurred through the altruistic sharing of equipment and the mentoring of novices, activities that fostered closer relationships among participants (20). One player described her experience as follows:

When I first arrived, I told the organizers, “I'll just watch for a while. I don't want to disturb them.” She quickly replied, “Wait, I'll grab you a skateboard.” As I was about to follow her, a girl nearby exclaimed, “I have two skateboards! You can use this one of mine.” After skating for less than 50 meters, another girl who was smoothly skating behind me commented, “You look like a snowboarder; your technique is off.” For the next half hour, a surfskate club member I had just met kindly taught me the fundamentals of powering a surfskate board. (Surfskate, ZL)

Finally, lifestyle sports place a greater emphasis on “being together,” unlike the overly competitive and institutionalized values typical of mainstream sports. Frisbee participants consistently expressed a preference for a dignified defeat that adheres to the spirit of the game over a dishonorable victory marked by tactical fouls. They cherish the thrilling moments of the game, appreciating not only their own team's efforts but also the sporting qualities and virtues of the opposing players. Together, these embody a spirit of tolerance, solidarity, and emotional connection with the sport's particular culture. As one frisbee player explained:

We're more focused on finishing the game amicably, which is a much bigger priority than just winning. When you make a good catch, whether it's your opponent or teammate, they'll compliment you with a “wow, nice catch!” The first response is always encouragement. Even if I drop the disc and we switch from offense to defense, no one blames anyone; instead, we all encourage each other to improve, admitting, “My mistake, I didn't make a good catch!” (Frisbee, LLY)

This ritualized interactive process, including rivalry recognition and active acknowledgment of mistakes, aims not to demonstrate superiority but to reinforce egalitarian principles. Sharing sports equipment between groups, engaging in common training sessions, and the mutual lending of equipment serve to articulate, maintain, and promote shared values and interdependence. These practices, driven by egalitarian power relations, are crucial factors in sustaining the participants' involvement.

### 4.4 Finding real people in the virtual age

With the recent advent of technologies such as immersive interaction, artificial intelligence, and advanced communication networks, social interactions spanning time and space—enabled by Internet technology—have become increasingly sophisticated. Social media is now integrated into nearly every aspect of daily life, connecting individuals and activities across various domains. Meanwhile, the “virtual presence” of the Internet is becoming increasingly prominent. Not only are individuals deeply embedded in cyberspace, but the Internet has also evolved into a primary spiritual space for contemporary youth. The prolonged use of online platforms and social networking apps is increasingly tying young people's time and energy to social media. As a result, many are experiencing social media fatigue and beginning to question the authenticity of their online interactions (49).

There are so many young people living the same life as me, and I don't want to stay home and just “lay around” anymore. I can hardly communicate with anyone! But now, with a different lifestyle, I can be with people, exercise together, share my feelings—I'm not alone anymore. (Surfskate, ZL)

In fact, these consumers care primarily about the instrumental meaning of lifestyle sports: social interaction.

Even though I've always lived here [in Xi'an], I started feeling disconnected, as if I'd lost my daily routines and interests. So I told myself, I need to go out and make real friends. Today, I can play flag football, and tomorrow I can go skating [i.e., surfskating] by myself. At this moment, it feels like all of these activities are made for me. (Surfskate, SX)

Lifestyle sports have become a unique tool for social interaction. First, regarding accessibility, the low entry barriers and diverse options make these activities widely popular and socially inclusive. The low learning curve and variety of choices make it easier for youth to engage in social interaction. Second, in terms of scheduling, unlike traditional sports that require full teams playing at fixed times, lifestyle sports can be played at any time. With active group chats on platforms like WeChat, participants can join or leave activities at their convenience, ensuring flexible social interaction. Young people can instantly organize sports activities, share experiences, exchange technical tips online, and then arrange offline meetups, continuously strengthening their social bonds. Compared to solely online communities, the communication and interaction among lifestyle sports enthusiasts are grounded in physical activity, making these activities more authentic and multidimensional. In contrast to traditional sports groups, the collectives that form around lifestyle sports offer more opportunities and spaces for interaction.

Likewise, lifestyle sports create a space for social interaction that fulfills the real social needs of youths.

When you play frisbee, you pass the disc, train, exchange tactics, travel together for tournaments, stay in hotels, visit venues, and explore cities together. In that environment, relationships are cultivated differently. You meet a lot of new people and get to know those living in the same area as you—what they're thinking, what they're doing, and the kind of people they are. (Frisbee, LW)

Young people evidently gain a greater sense of participation and initiative when they take up lifestyle sports. The interactive experiences gained from this social space quickly activate psychological projection and compensation mechanisms, providing a sense of fulfillment through social participation and a sense of belonging within the group.

## 5 Discussion

### 5.1 Understanding contemporary youth consumption traits from lifestyle sports

The consumption concept reflects individual views on various aspects of consumption, including modes, levels, orientations, and values (50). Lifestyle sports consumption represents a typical form of spiritual and cultural consumption that not only indicates a significant shift in the consumption structure but also demonstrates effective leadership in responding to changes in demand levels and structural optimization (51). Based on this point, the diversified qualities of the consumption philosophy of contemporary youth can be better understood.

Firstly, the consumer experience is prioritized, which encompasses the realization of the subject matter and the emotions elicited by sensory or psychological experiences. In the context of lifestyle sports consumption, youths pursue aims that extend beyond technical mastery and physical challenges to include visual enjoyment, mood enhancement, sensory comfort, and psychological alignment. In a China Youth Daily survey, 86.6% of the respondents expressed a willingness to spend more money to obtain superior experiences, with technical content (49.9%) and novelty and fun (48.9%) being their primary concerns (52). Emphasizing experience means focusing on the positive emotional feelings imbued during the consumption process. Therefore, contemporary young people are willing to invest in activities that captivate their interest and provide novelty, creativity, and advanced technological features. In lifestyle sports such as ultimate frisbee, surfskate, or rock climbing, the interactive experience, competitive edge, team atmosphere, and equipment exemplify the typical consumption demands of these experiences.

Secondly, young people are increasingly rejecting extravagant consumption in their symbolic consumption; instead, they are favoring cost-effective, environmentally friendly alternatives. In 2021, Economic Daily announced five annual trending phrases in the field of Chinese consumption, with “consumption of an affordable alternative” appearing on this list (53) The phrase refers to substitute brands or products that deliver satisfactory services or functions but cost significantly less. In 2024, the Beijing News published the Shell Financial 2024 Chinese Youth Consumption Trend Report (54). This document highlighted a distinct preference among young Chinese consumers, particularly those born in the 1990s and 2000s, for more cost-effective products. Over 40% of these younger consumers reported a tendency to compare prices, demonstrating a clear preference for lower-priced commodities while shopping. This trend is evident in lifestyle sports, where there is less emphasis on luxury brands and more on understated coolness. Participants showcase their style and taste without flaunting wealth; they focus on authenticity and individuality rather than following trends or displaying ostentation. In a sense, it is precisely this concept that aligns lifestyle sports with Social Practice Theory, which emphasizes routine and everyday behaviors grounded in not the fleeting appeal of luxury consumption but sustained engagement and participation. In this context, engaging in lifestyle sports becomes a continuous, repeatable practice that underscores the sustainability and intrinsic value of consumption.

Thirdly, symbolic consumption is embraced. Aspects of lifestyle sports, such as wearing favored attire, capturing timely photographs, and enjoying runs with friends, serve as both leisure activities and a means of symbolic consumption. While participants use these activities to distinguish their tastes and identities, they also gain ongoing satisfaction from sharing these experiences on social media. Furthermore, young people's engagement with symbolic consumption does not imply their blind adherence to symbols; instead, it signifies their intention to craft fashion labels, consumption concepts, youth symbols, and lifestyles that reflect their unique subcultural traits. Although Social Practice Theory critiques interpretive approaches that overemphasize the symbolic dimensions of consumption (55), symbolic consumption remains a salient feature in the lifestyle sports practices of Chinese youth. Unlike Thorstein Veblen's notion of “conspicuous consumption,” this engagement does not involve passive imitation. Rather, it reflects a conscious and intentional process through which young people construct fashion labels, consumption values, youth symbols, and lifestyles that express their distinct subcultural identities.

Young people's consumption concepts have evolved alongside societal changes, becoming increasingly diverse over time. On one hand, caution is required when examining the alienation of consumption concepts, which can manifest as over-consumption, status-seeking, and the uncritical consumption of trending content and knowledge under the influence of digital technology. On the other hand, leveraging contemporary youth consumption trends might rejuvenate traditional sports and invigorate youth consumption power.

### 5.2 The social value of lifestyle sport: enhancing social trust and embracing a sport-centric lifestyle

In lifestyle sports, the enhancement of social trust emerges as a key benefit. This consumption practice encourages participants to escape their self-imposed confines, fostering trust through cooperation, encouragement, and engagement with teammates. Such players evidently tend to prioritize emotional sharing over strategic interest-based interactions. This is reflected in their sincere expression of opinions, intentions, emotions, and desires, which are manifested through online communication and information sharing, as well as offline interactions marked by care, encouragement, and appreciation. Under the pervasive influence of commercialism, traditional sports have come to be regarded as elitist and extravagant, having distanced themselves from everyday life and transformed themselves into arenas dominated by commercial interests (56). In contrast, lifestyle sports foster youth interaction in intimate settings, where emotions represent not tools for strategic manipulation but a means to enhance genuine, interest-free connections.

Schatzki (57) views subjective elements—such as purpose, beliefs, and emotions—as relational components within practice. He argues that once a practice is established, these elements no longer belong to the individual but become intrinsic to the practice itself. Thus, as non-elitist forms of practice, lifestyle sports reinforce young people's commitment to community and social trust through emotional exchange and a shared sense of identity (58). They serve as vehicles for social practice, enabling individuals to engage with collective life while also promoting social order and group cohesion. The development of Olympic skateboarding talent in the UK is rooted in local skate parks, promoting a broader, non-competitive spirit. This phenomenon extends beyond the UK: it became a major feature of the women's skateboarding event at the 2020 Olympics (59), and it is now also evident in Asia. National and international coaches must recognize the importance of this mutual supportive spirit for the mental health and character development of today's youth; this spirit should continue to be nurtured in their charges.

Additionally, the trend of pursuing lifestyle sports is becoming increasingly apparent in China, where engaging in sports and leisure activities has evolved into a significant aspect of daily life. The essence of living through sports is to provide everyone with a sense of freedom and pleasure in their daily routines (60). Lifestyle sports facilitate access to leisure, likely driven by the reinforcement of trust and a profound “emotional orientation” that proves more motivating than a “material orientation.” Therefore, the national promotion and development of lifestyle sports should extend beyond economic considerations and competitive values to embrace the inclusiveness, community spirit, and shared emotional identities inherent in various sports cultures. It is vital to recognize the multifaceted roles of lifestyle sports in shaping social relationships and the spiritual lives of contemporary youth. Emphasizing the emotional orientation and personal aspects of lifestyle sports would foster not only respect for individuality but also an internal awareness of equality and reciprocity within the movement. This approach would significantly enhance the social value of lifestyle sports, contributing to the construction of social civilization in the new era.

## 6 Conclusion

In this study, the dynamic intersection of lifestyle sports and youth consumer behavior in China is explored, highlighting how lifestyle sports are not merely activities; they also play significant roles in the lives, emotions, and social interactions of Chinese youths.

The findings indicate several points. First, lifestyle sports consumption focuses on actual experiences of a sport, which are used to find oneself and express freedom. Second, group-based consumption emphasizes the role of sport in fostering communal and emotional connections through shared experiences, with sports acting as catalysts for forming “neo-tribes”. These are temporary, fluid communities driven by shared enthusiasm and emotional resonance. Thirdly, lifestyle sports continue to hold the meaning of symbolic consumption and aim to generate differentiation, influenced significantly by Internet media. The last point refers to the consumption of lifestyle sports as tools and spaces for socialization.

In conclusion, the popularity of lifestyle sports among Chinese youths reflects broader societal transformations concerning individuality, community, and consumption. However, certain limitations of this analysis should be acknowledged. Future researchers should consider the experiences and attitudes of youths in rural or less urbanized areas, where access to and perceptions of lifestyle sports may differ markedly. Additionally, a broader quantitative study could explore the impacts of economic constraints, educational levels, and family backgrounds on the consumption and participation in lifestyle sports, providing a more comprehensive understanding of the national landscape.

## Data Availability

The original contributions presented in the study are included in the article/Supplementary Material, further inquiries can be directed to the corresponding author.

## Footnotes

^1^A mainstream social media platforms in China, primarily targeting urban middle-class female users.
